# Supplementary figures and images for: Fragment-based drug discovery for transthyretin kinetic stabilisers using a novel capillary zone electrophoresis method
Source: PLoS One. 2025 May 14;20(5):e0323816. doi: 10.1371/journal.pone.0323816 (PMC12077799; doi:10.1371/journal.pone.0323816)

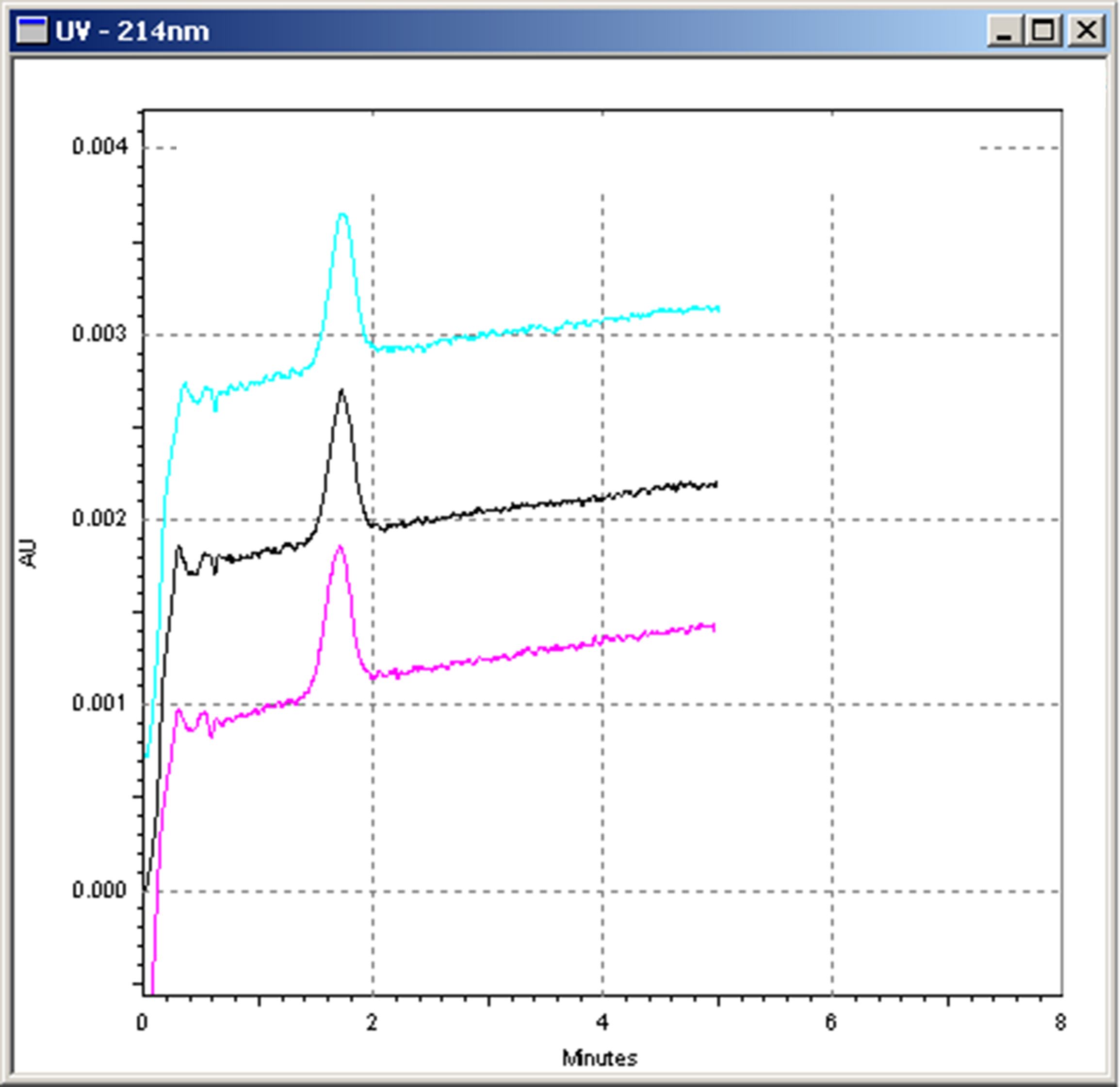

Supplement: S1 Fig — Inject Buffer containing 2 μM of transthyretin diluted in Buffer A was prepared. A small volume was injected and separated in a capillary filled with Buffer A. The process was repeated three times with the same capillary and electrophoretic conditions (coated capillary, voltage 30 kV, outlet pressure injection 0.5 psi for 5 seconds). The three electropherograms observed were virtually identical in terms of peak migration time, height and area. This excellent reproducibility and unmatched separation efficiency (even for macromolecules in this case) has made CZE an invaluable technique in fragment screening assays as well as protein analysis. (TIF) [file pone.0323816.s001.tif]

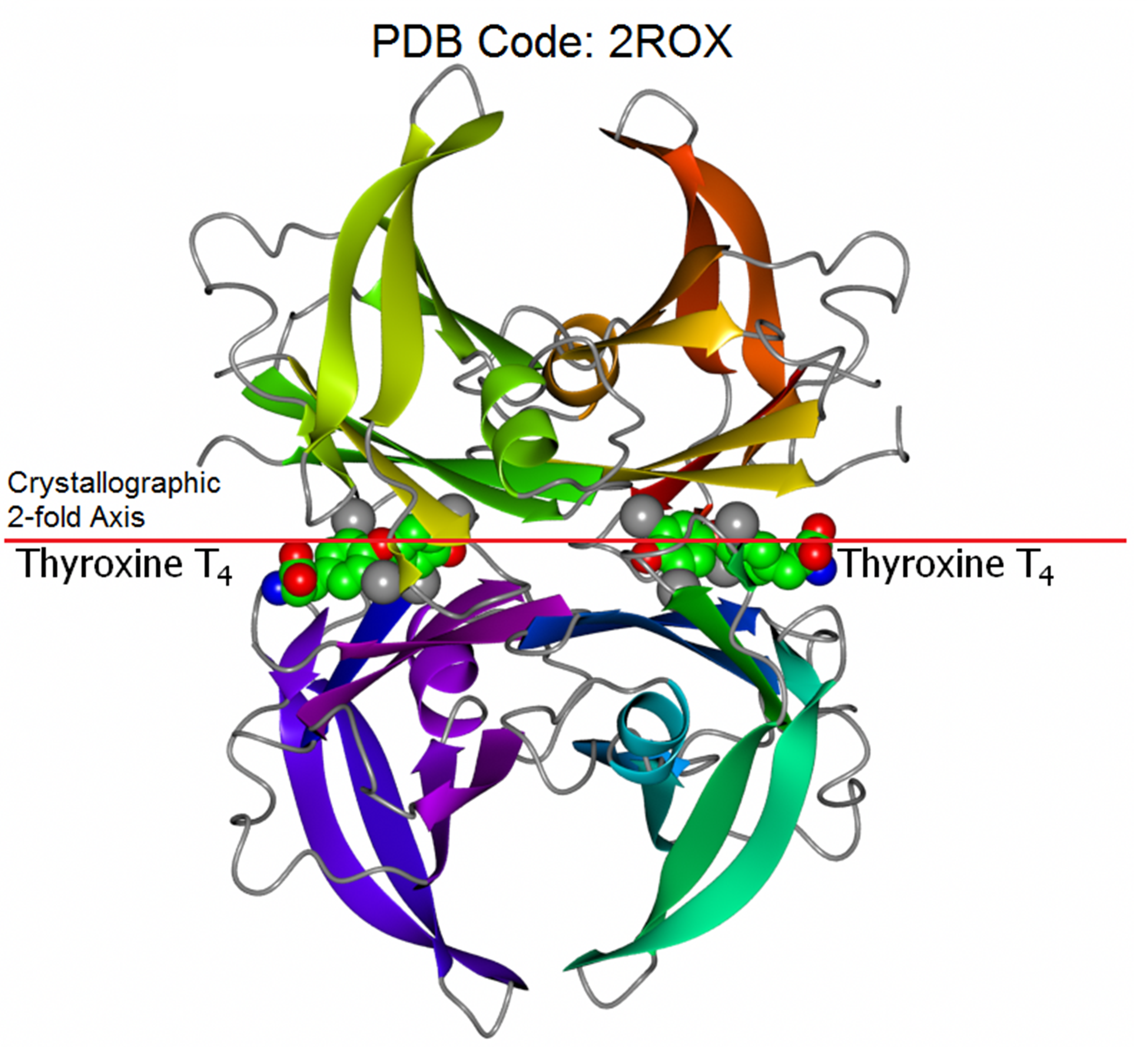

Supplement: S2 Fig — A ribbon representation of transthyretin homotetramer bound to Thyroxine T4. There are two structurally identical thyroxine binding sites in between the dimer-dimer interface with a line of 2-fold symmetry running across the binding channel (red line). The overall shape of a TTR tetramer resembles of an hourglass and each monomer makes up half of a binding site. Beta-sheet is frequently observed in transthyretin and dissociation of the tetramer can lead to formation of amyloidogenic monomers. Binding of thyroxine or other ligands could stabilise the TTR tetramer by forming non-covalent bonds to each monomer, effectively “securing” them in position. (TIF) [file pone.0323816.s002.tif]

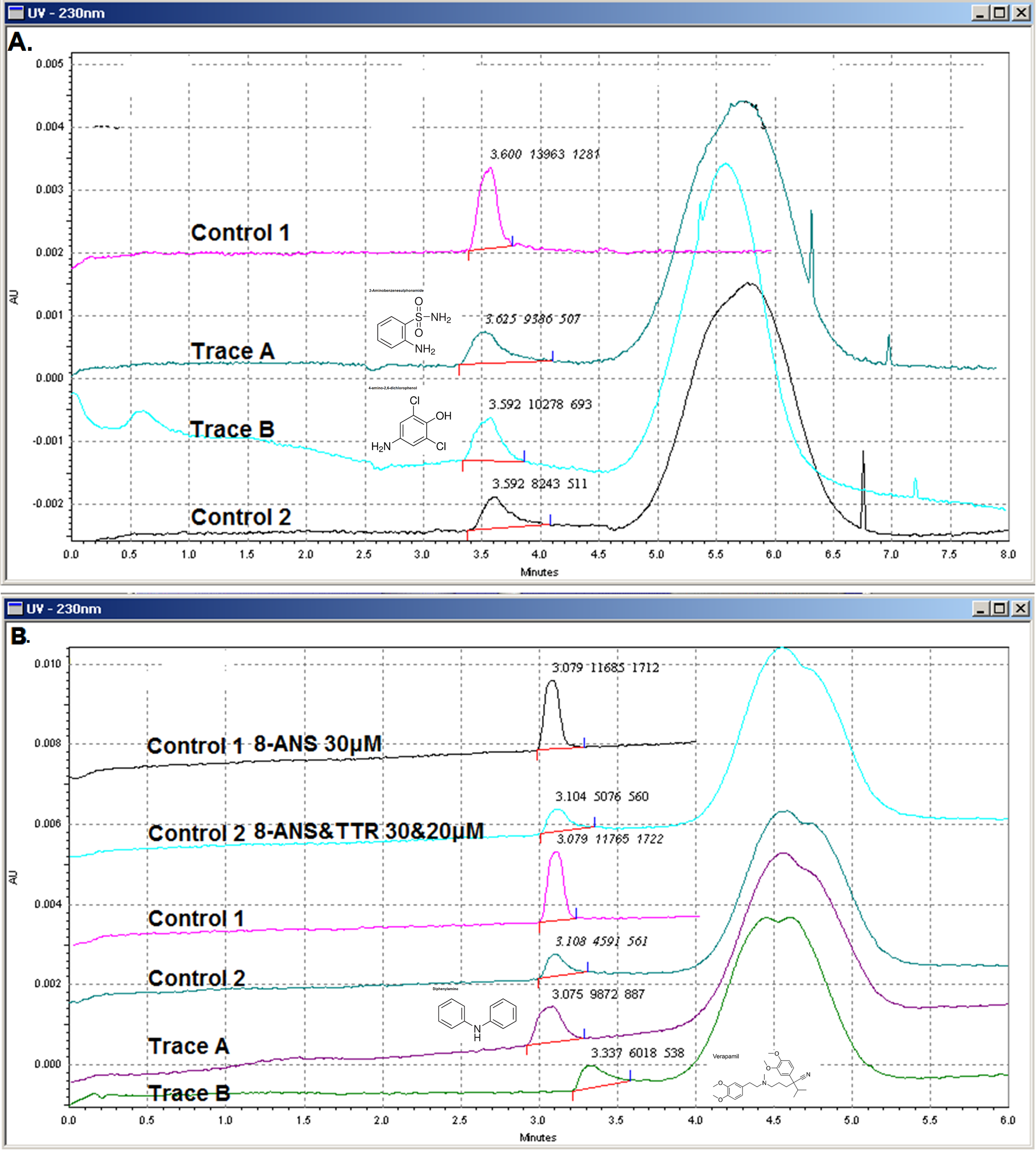

Supplement: S3 Fig — (A.) A fixed volume of Control 2 was injected and separated in a capillary filled with Running Buffer containing different compounds. 500 μM of 2-Aminobenzensulphonamide (Trace A) caused no change in free 8-ANS peak height. 200 μM of 4-Amino-2,6-Dichlorophenol (Trace B) has clearly displaced some 8-ANS from TTR. (B.) A fixed volume of Control 1 and Control 2 was injected and separated in a capillary filled with Buffer C twice resulting in identical peaks. Free 8-ANS peak height increased significantly when 250 μM of Diphenylamine was added to the Running Buffer (Trace A), but not with 300 µ M of Verapamil (Trace B) a negative. Numeric values atop free 8-ANS peaks indicate migration time (minutes), peak area and height respectively. (TIF) [file pone.0323816.s003.tif]

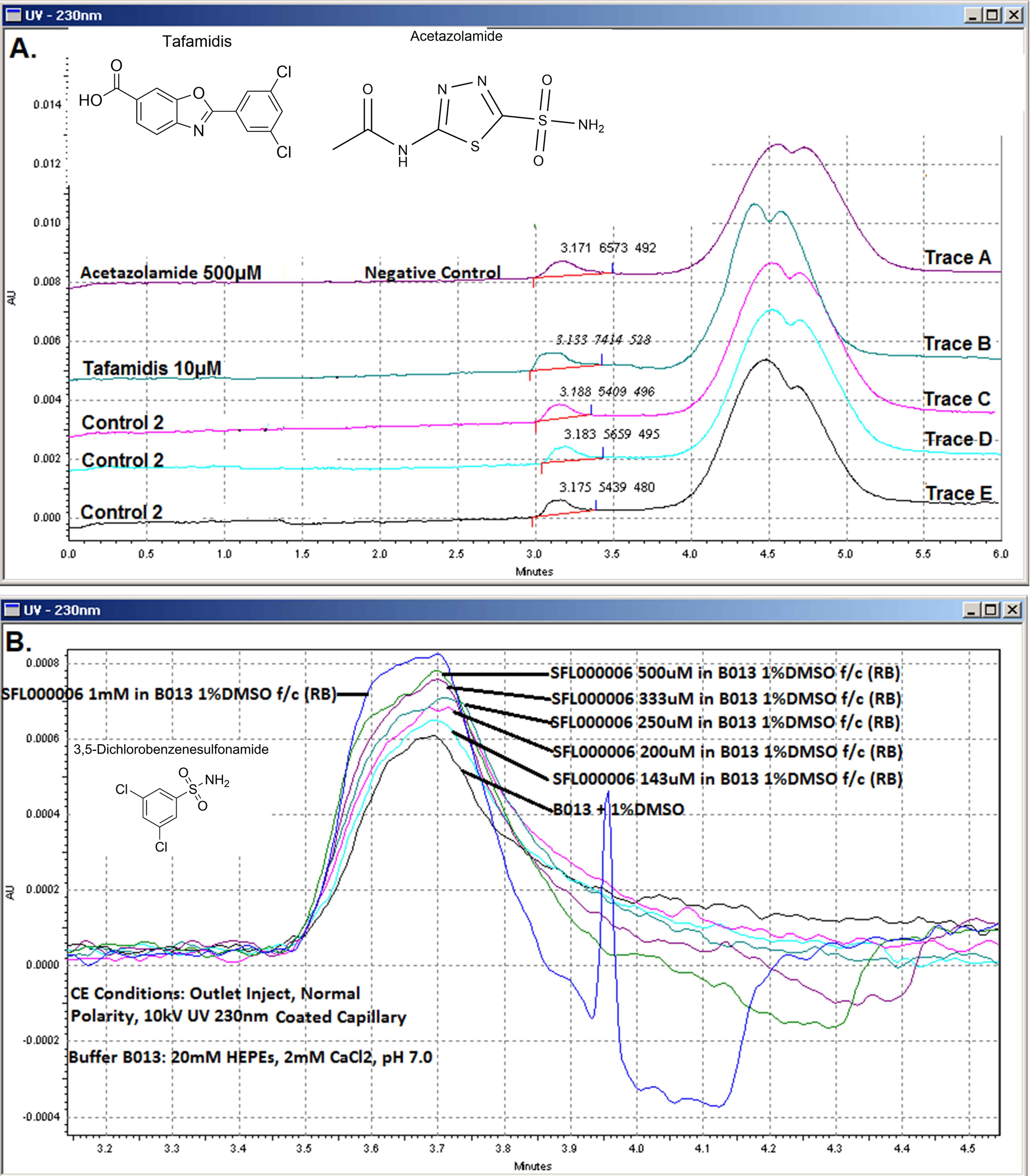

Supplement: S4 Fig — (A.) A fixed volume of Control 2 was injected and separated in a capillary filled with Buffer C three times (Trace C-E) with highly consistent free 8-ANS peak migration time, area and height. Different compounds were introduced into the Running Buffer. A significant increase in free 8-ANS peak height compared to Control 2 did not happen with 500 µ M Acetazolamide (Trace A) but was achieved by Tafamidis at 10 µ M (Trace B). Numeric values atop free 8-ANS UV peaks indicate migration time (minutes), peak area and peak height respectively. (B.) A sequence of CZE separations was performed with increasing concentrations of 3,5-Dichlorobenzenesulphonamide (SFL000006) added to the Running Buffer. Free 8-ANS UV peak from all traces were superposed and zoomed in omitting the absorbance peak of TTR. Free 8-ANS peak height increased as more SFL000006 was added to the Running Buffer. There was a sharp peak at 3.95 minutes in the blue trace when the compound concentration was at 1 mM. This was treated as a serendipitous spike, possibly due to high compound concentration. (TIF) [file pone.0323816.s004.tif]

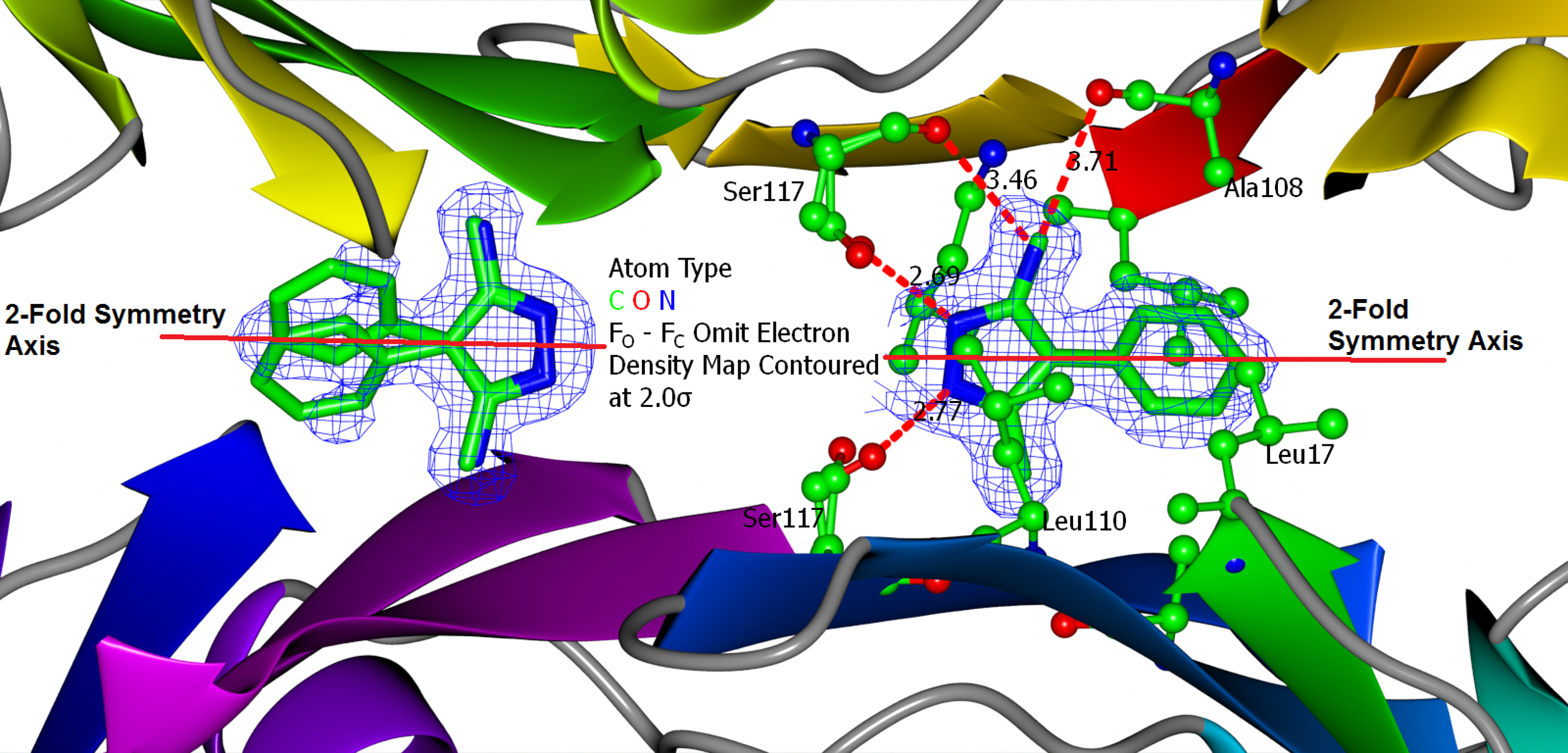

Supplement: S6 Fig — Binding of SFL001561 to TTR involved a double hydrogen bonding system between the pyrazole ring and the two Ser117 sidechains. This interaction is essential to binding affinity because a methyl substitution on one of the pyrazole nitrogen atoms (SFL001562) had decimated assay activity (S2 Table). The methyl and phenyl substituent contribute to the ligand’s affinity by hydrophobic contact with Leu110 and Leu17 sidechains. There are two possible hydrogen bonds between the amino group and the carbonyl oxygen of Ser117 and Ala108, which is something not seen on previously studied TTR ligands. The binding mode of SFL001561 between the two sites was different, albeit not as obvious as seen for SFL001535. In one site, the phenyl ring is slightly titled along the 2-fold axis compared to the other, another ligand-based structural evidence for binding cooperativity. Numbers near dashed red lines indicate bond length in Angstroms. (TIF) [file pone.0323816.s006.tif]
