# Supplementary material for: Fragment-based drug discovery for transthyretin kinetic stabilisers using a novel capillary zone electrophoresis method
Source: PLoS One. 2025 May 14;20(5):e0323816. doi: 10.1371/journal.pone.0323816 (PMC12077799; doi:10.1371/journal.pone.0323816)

S5 Fig. TTR Co-Crystal Structure of DIA, DSF and NPA


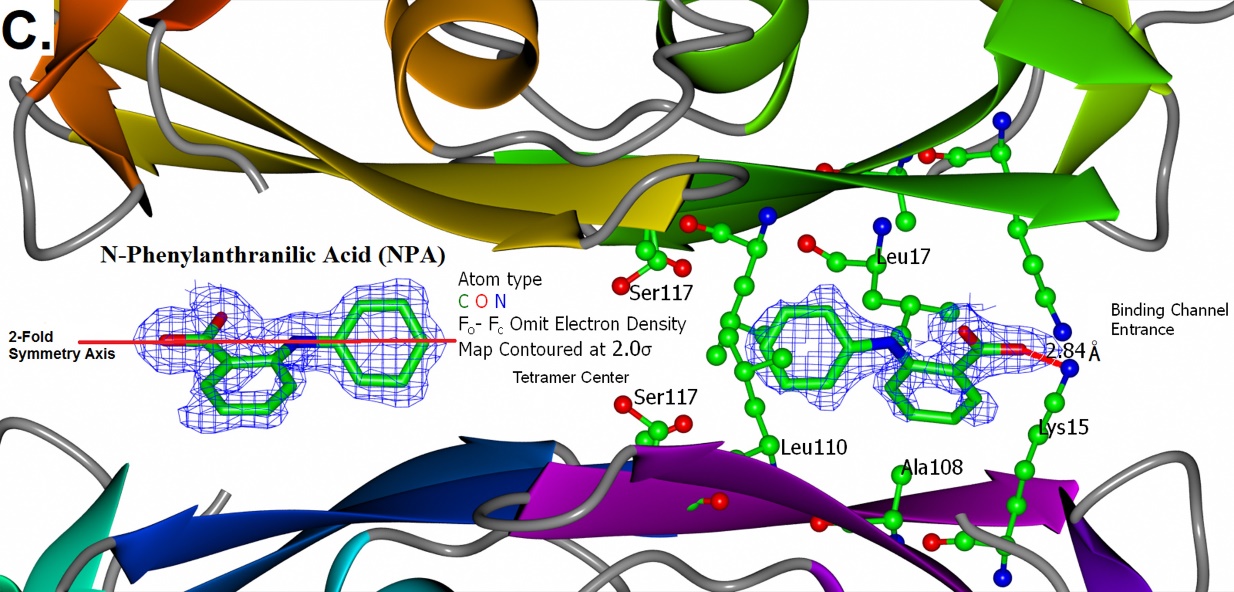

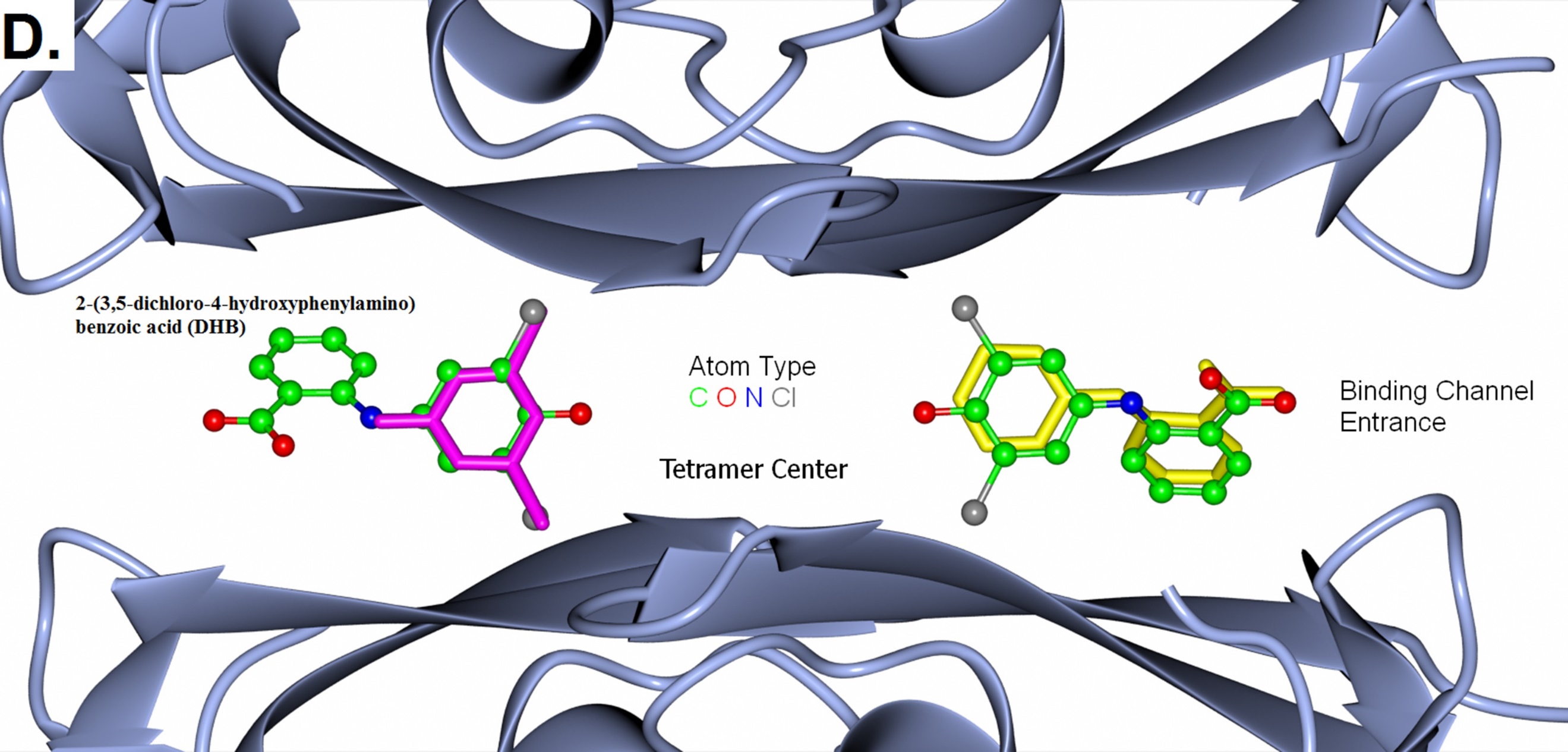

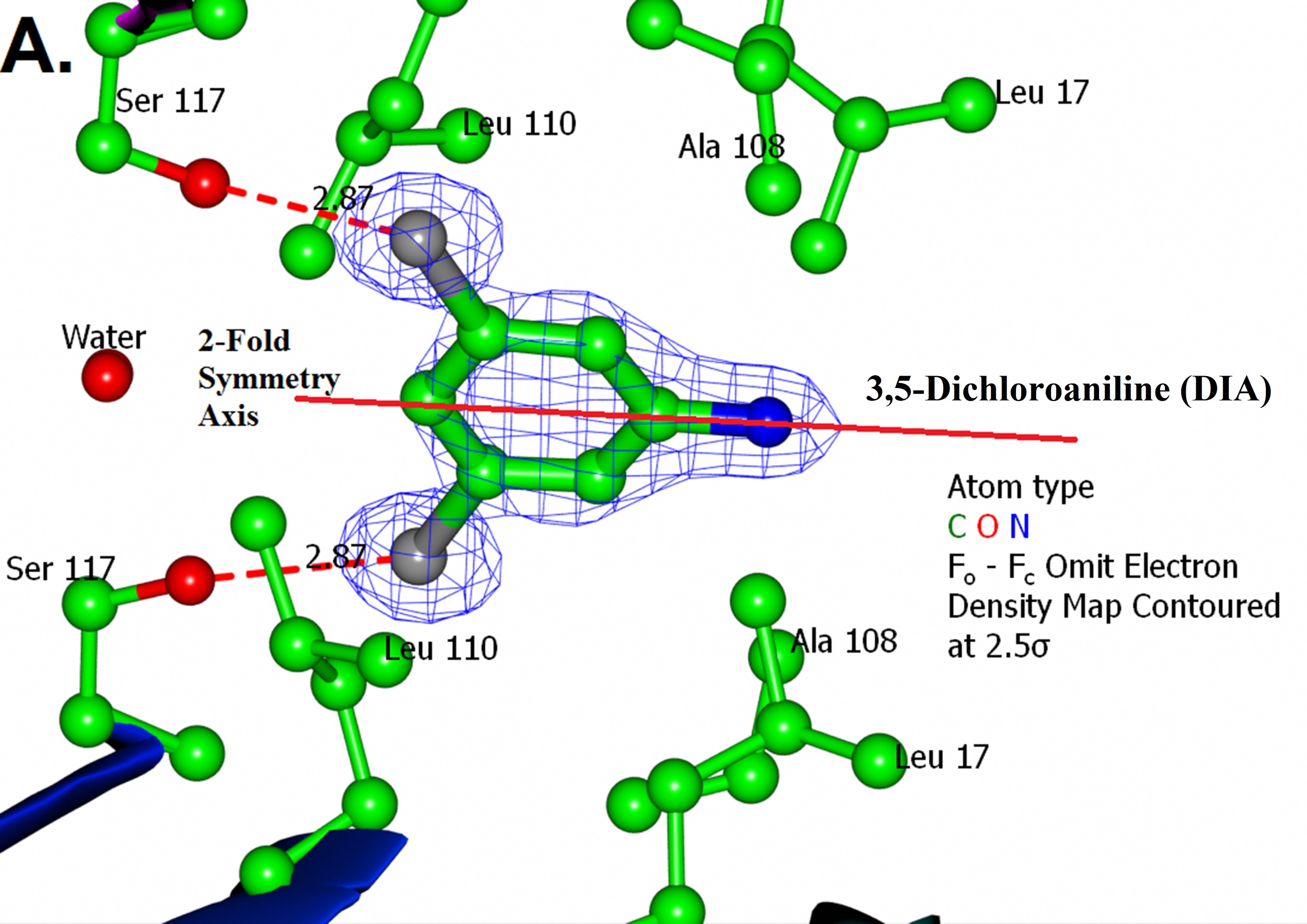


(**A.**) The chlorine atoms of 3,5-Dichloroaniline (DIA) are buried deep inside the binding channel and have formed halogen bonds to each of the Ser117 sidechains nearby. (**B.**) A chemical switch from amino to sulphonamide has caused the dichlorobenzene to face the opposite direction towards the binding channel entrance. (**C.**) Unlike DIA and DSF, N-Phenylanthranilic Acid (NPA) occupied both sites in the binding channel. Its diphenylamine is wrapped around by the sidechains of Leu110 and Leu17 whilst the carboxylate forms ionic interaction with Lys15. (**D.**) The crystal structures of DIA (magenta cylinders), NPA (yellow cylinders) and DHB (balls & sticks) in complex with TTR are superposed by Secondary Structure Matching. The corresponding atomic coordinates between these three compounds matched accurately. Numbers near dashed red lines are bond lengths in Angstroms.


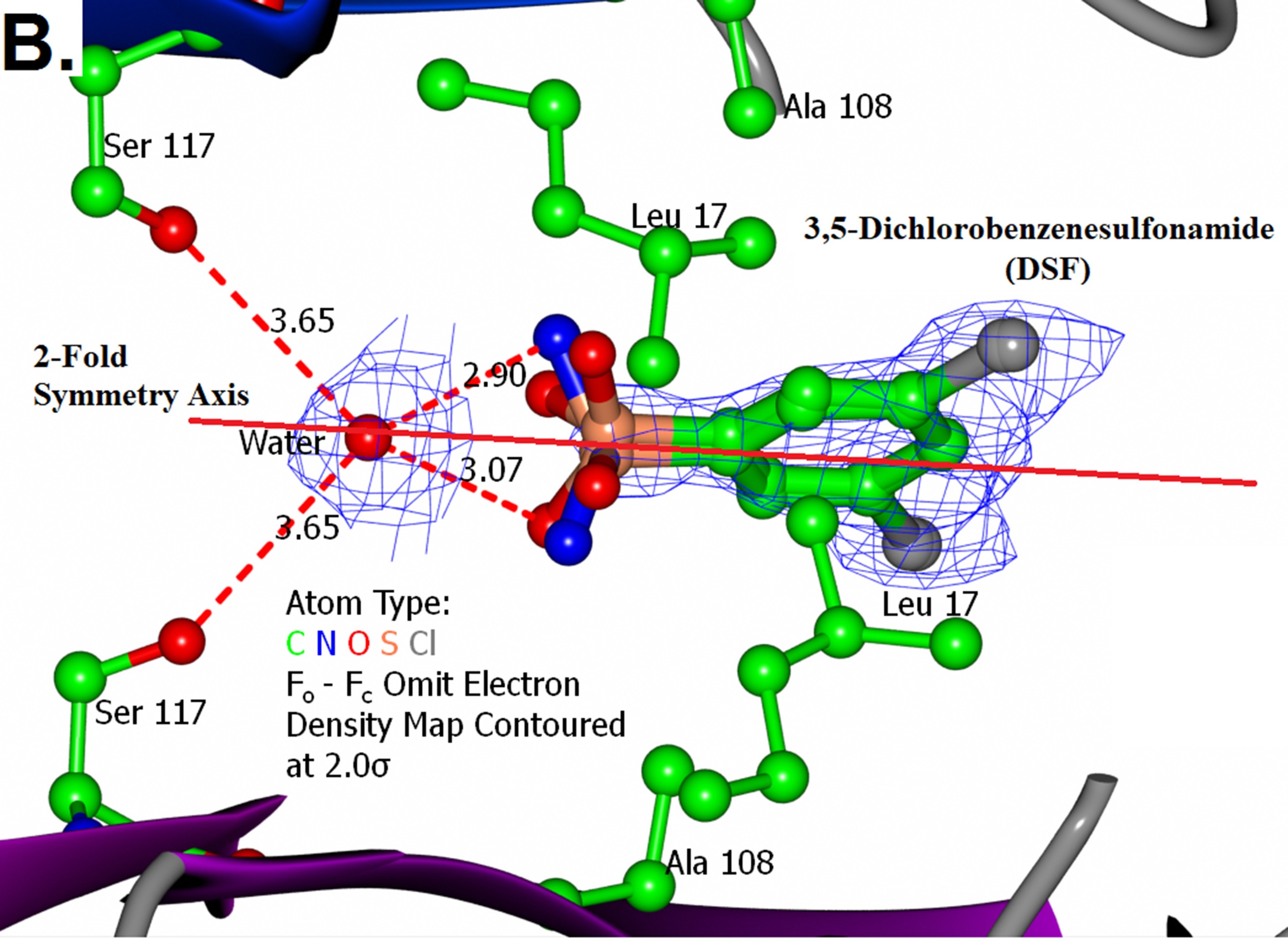

Supplement: S5 Fig — (A.) The chlorine atoms of 3,5-Dichloroaniline (DIA) are buried deep inside the binding channel and have formed halogen bonds to each of the Ser117 sidechains nearby. (B.) A chemical switch from amino to sulphonamide has caused the dichlorobenzene to face the opposite direction towards the binding channel entrance. (C.) Unlike DIA and DSF, N-Phenylanthranilic Acid (NPA) occupied both sites in the binding channel. Its diphenylamine is wrapped around by the sidechains of Leu110 and Leu17 whilst the carboxylate forms ionic interaction with Lys15. (D.) The crystal structures of DIA (magenta cylinders), NPA (yellow cylinders) and DHB (balls & sticks) in complex with TTR are superposed by Secondary Structure Matching. The corresponding atomic coordinates between these three compounds matched precisely. Numbers near dashed red lines are bond lengths in Angstroms. (DOCX) [file pone.0323816.s005.docx]
