# Supplementary material for: Fragment-based drug discovery for transthyretin kinetic stabilisers using a novel capillary zone electrophoresis method
Source: PLoS One. 2025 May 14;20(5):e0323816. doi: 10.1371/journal.pone.0323816 (PMC12077799; doi:10.1371/journal.pone.0323816)

S2 File. Free 8-ANS Peak Height Restoration Titrations from 14 Fragment Hits


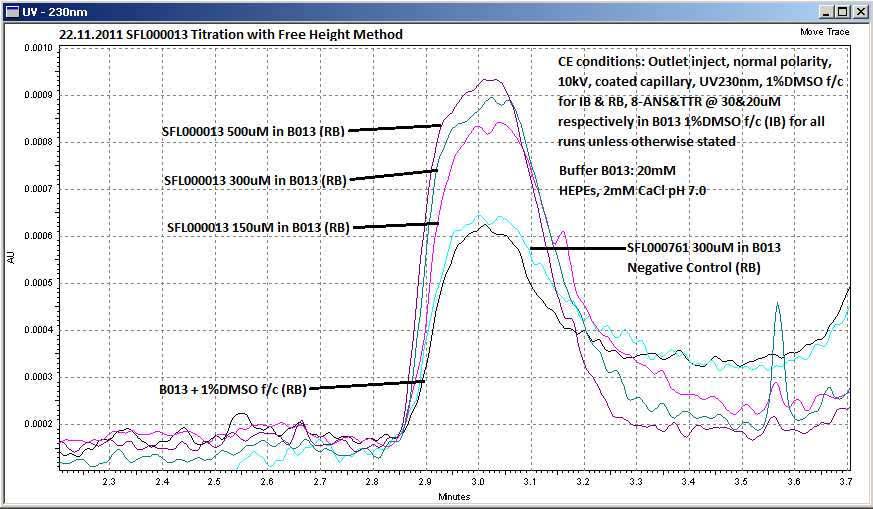

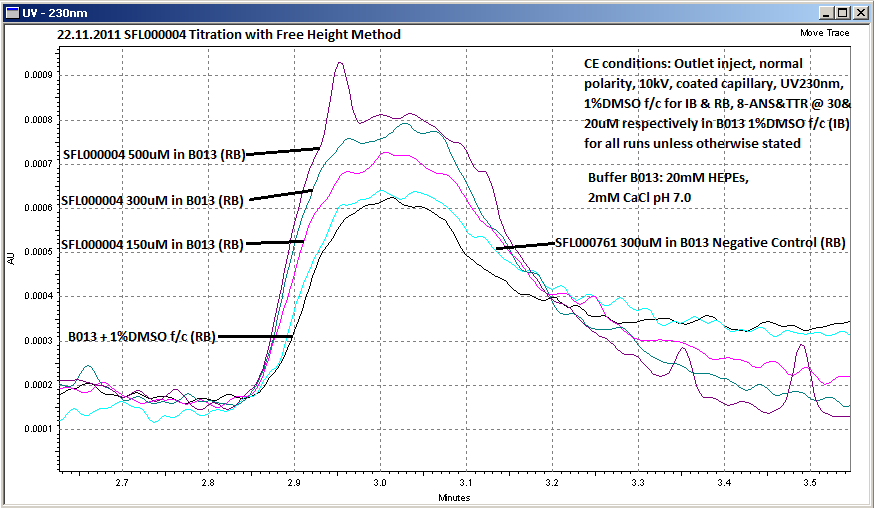

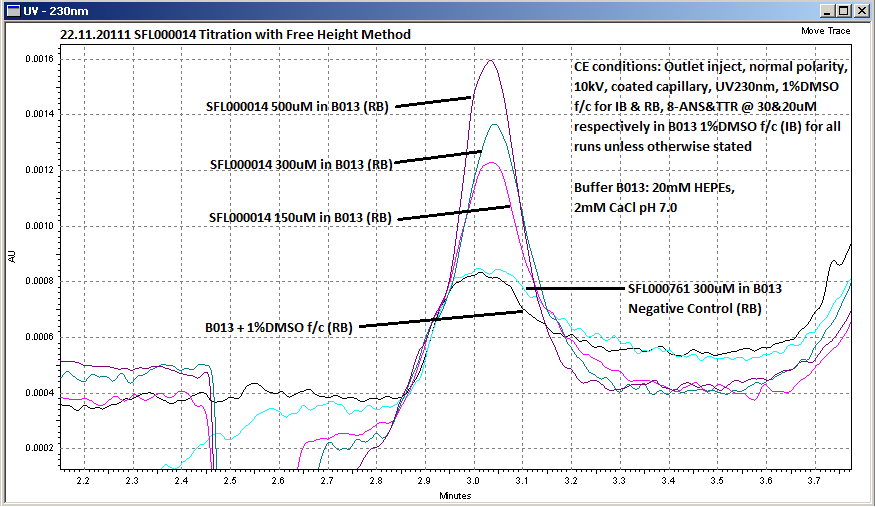

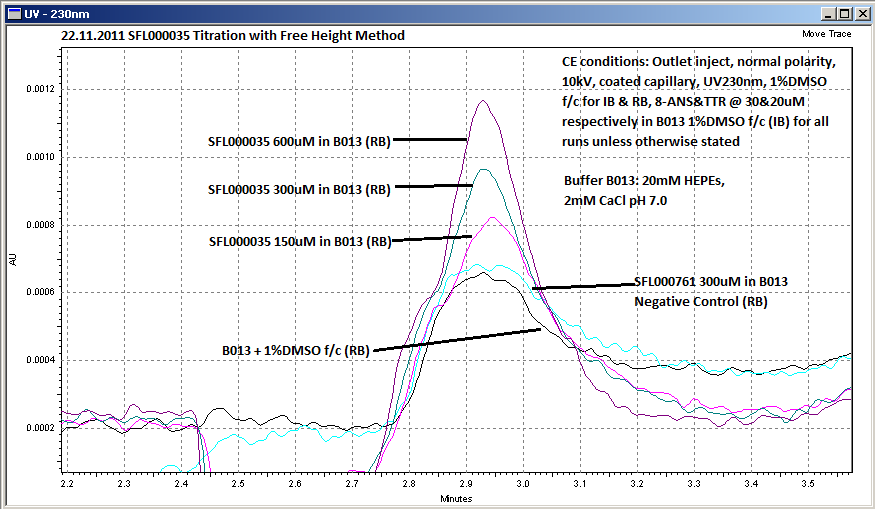

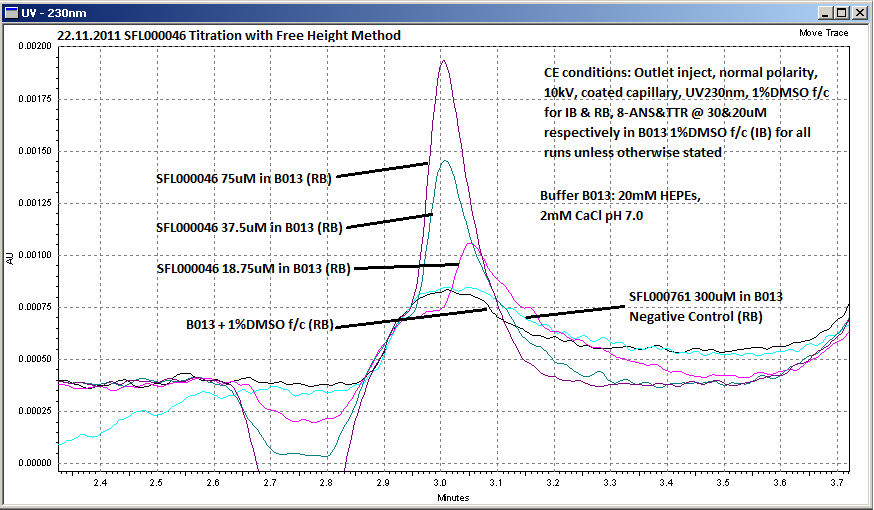

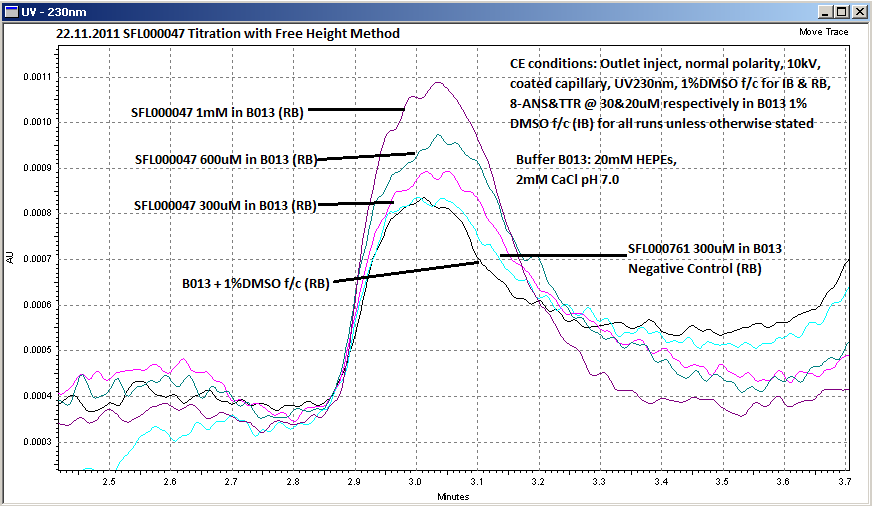

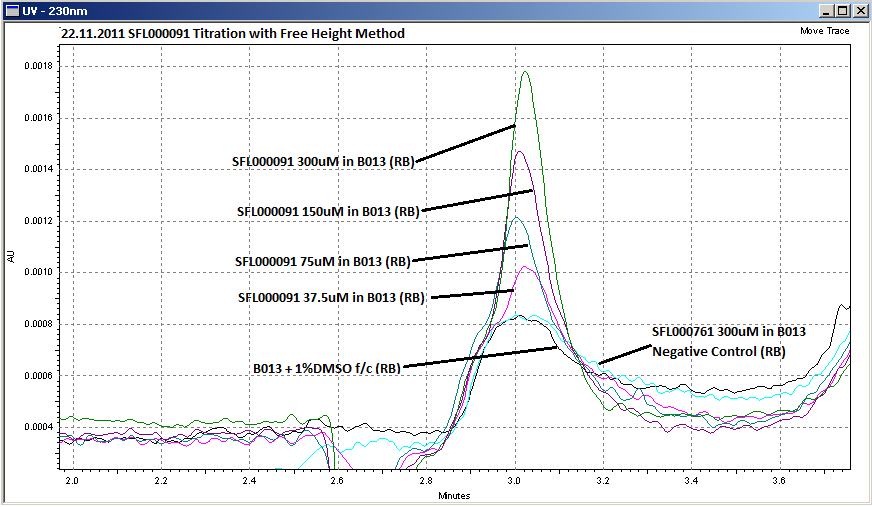

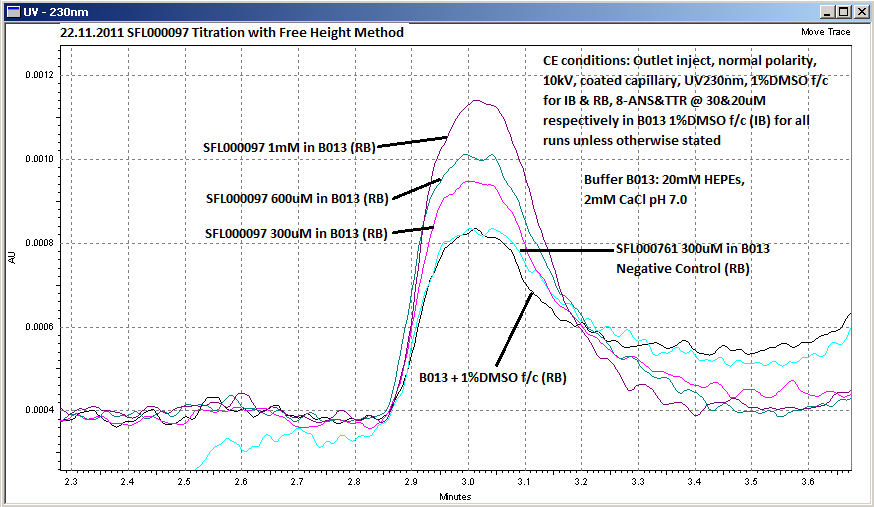


Multiple sequences of CZE separations were performed with increasing concentrations of fragment hits added to the Runing Buffer. Hit titration concentrations ranged from 18.75 µM up to 1 mM depending on 8-ANS displacement potency. Free 8-ANS UV peak from all traces were superposed and zoomed in omitting the absorbance peak of TTR.


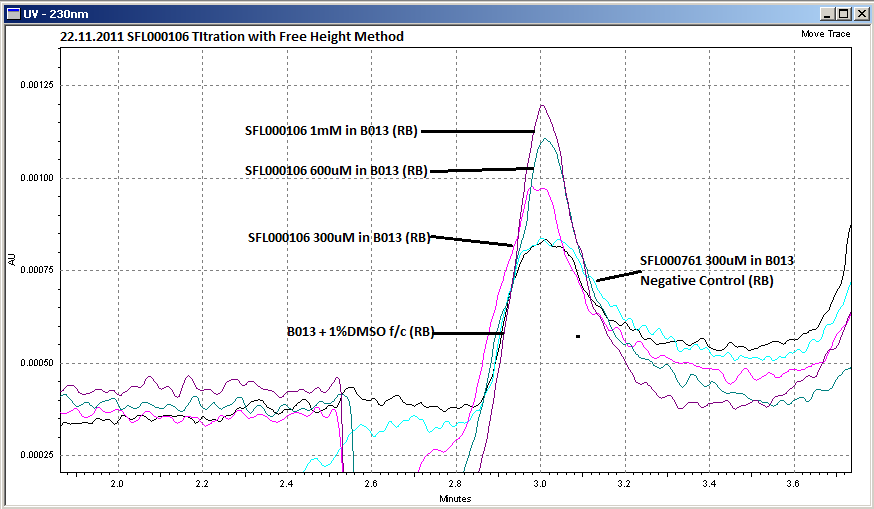

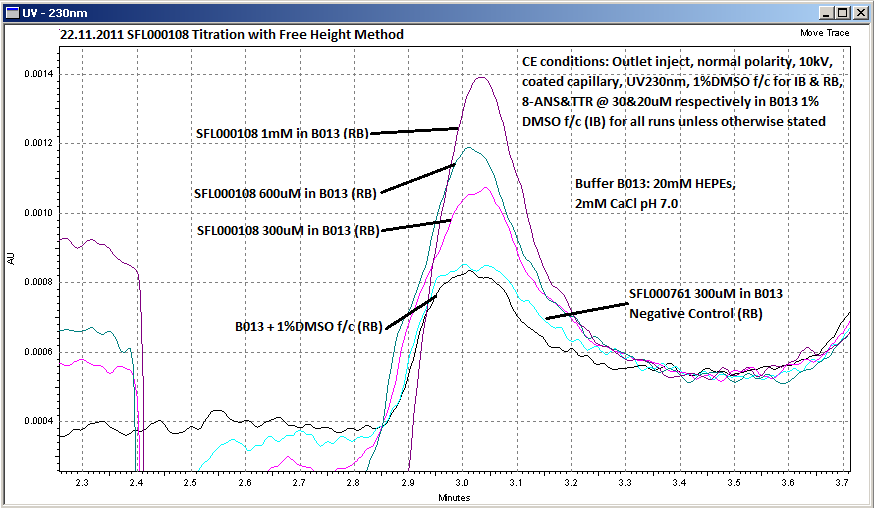

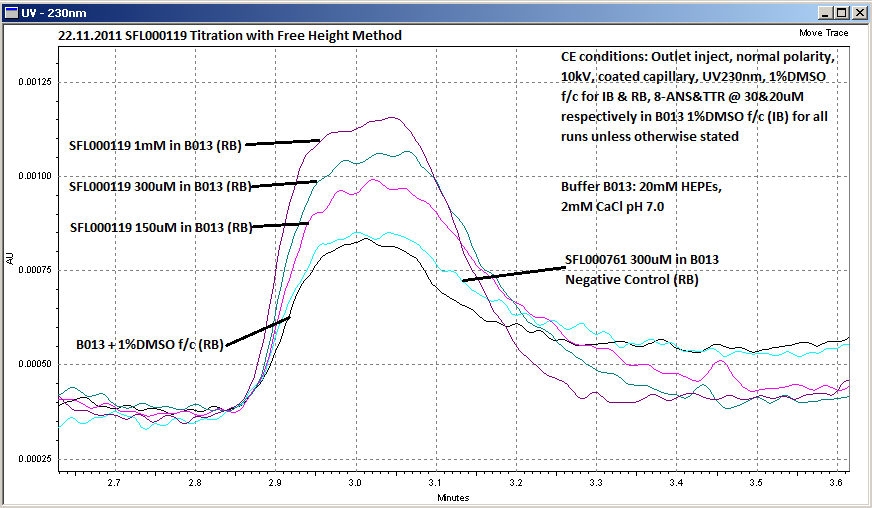

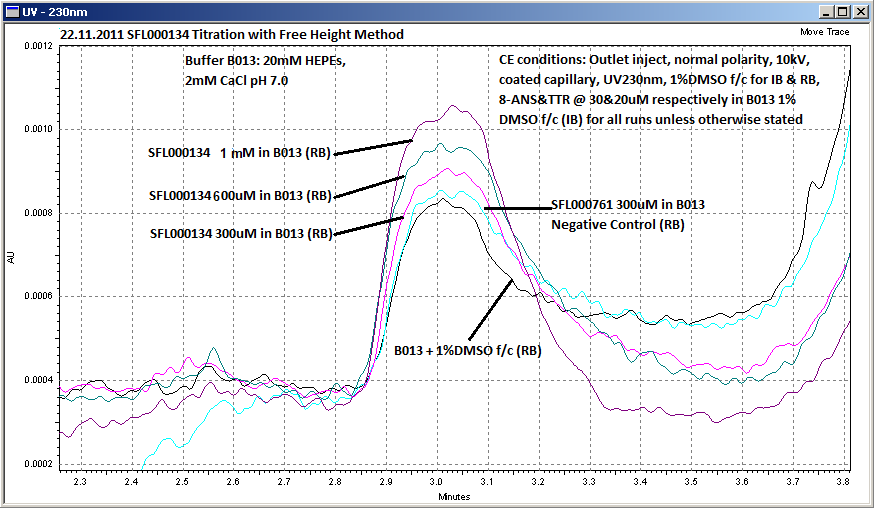

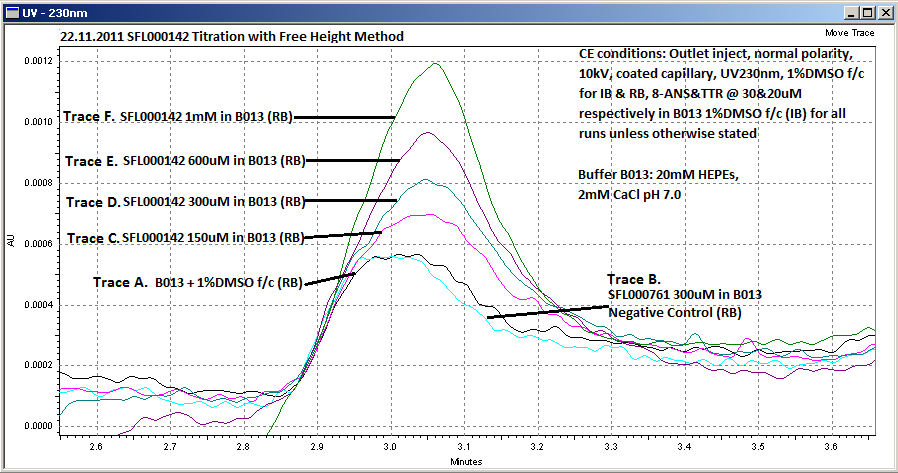

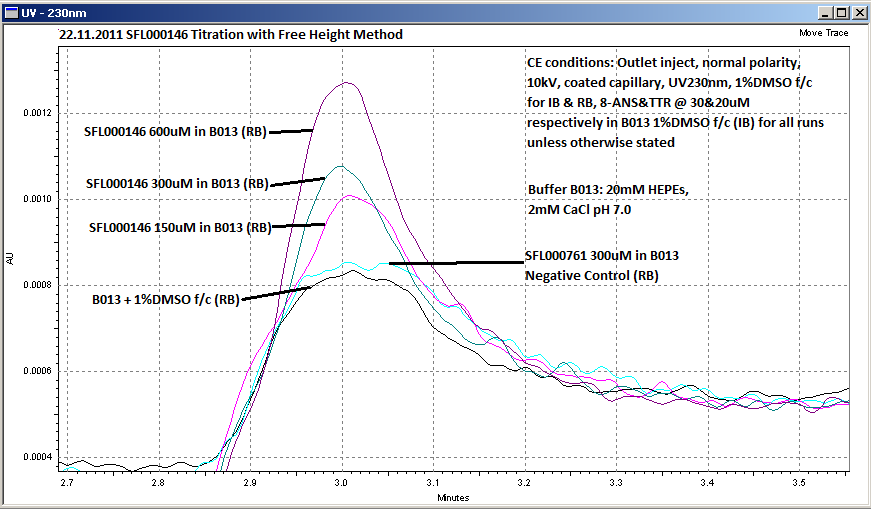

Supplement: S2 File — (DOCX) [file pone.0323816.s012.docx]
